# Evaluating Starter Feeding on Ruminal Function in Yak Calves: Combined 16S rRNA Sequencing and Metabolomics

**DOI:** 10.3389/fmicb.2022.821613

**Published:** 2022-06-06

**Authors:** Yin Wang, Hongze Xia, Qien Yang, Deyu Yang, Shujie Liu, Zhanhong Cui

**Affiliations:** ^1^Qinghai Academy of Animal Science and Veterinary Medicine, Qinghai University, Xining, China; ^2^Northwest Institute of Plateau Biology, Chinese Academy of Sciences, Xining, China

**Keywords:** feeding strategies, ruminal development, ruminal microbiota, ruminal metabolomics, yak calves

## Abstract

For young ruminants, starter feeding can effectively facilitate the growth and development of rumen in ruminants, but the development of rumen is an important physiological challenge as it remains unclear for the mechanism of starter feeding stimulating. In this study, we performed an analysis of ruminal microbiota and their metabolites in yak calves to explore how the ruminal microbiota and their metabolites stimulate the ruminal function. This study associated 16S rRNA sequencing with liquid chromatography-mass spectrometry (LC-MS)-based metabolomics to evaluate the effects of starter feeding on ruminal microbiota diversity and metabolites in yak calves. We designed the experiment using 20 yak calves that were assigned equally into 2 groups, based on feeding milk replacer; the control (RA) group was fed with alfalfa hay while the treatment (RAS) group was fed with alfalfa hay and starter. After the experiment, we investigated the ruminal microbiota and metabolites through 16S rRNA sequencing and LC-MS-based metabolomics. During the preweaning period, the RAS group significantly promoted the growth performance and ruminal development in yak calves, including increases in body weight, chest girth, and development of rumen (*P* < 0.05). The RAS group increased the relative abundance of *Bacteroidota*, *Proteobacteria*, *Chloroflexi, Synergistota*, and *Spirochaetota* and decreased the abundance of *Firmicutes*, *Desulfobacterota*, *Actinobacteriota*, and *Actinobacteriota* at the phylum level (*P* < 0.05). At the genus level, the ruminal content of the RAS group was significantly enriched for *Rikenellaceae_RC9_gut_group* and *Ruminococcus*, while depleted for *Prevotella*, *Christensenellaceae_R-7_group*, and *NK4A214_group* (*P* < 0.05). A total of 37 metabolites were identified between the RA group and the RAS group, of which 15 metabolites were upregulated and 22 metabolites were downregulated compared with the RA group. Metabolic pathway analyses indicated that upregulated the metabolites of the RAS group yak calves were related to carbohydrate metabolism, ubiquinone, and other terpenoid-quinone biosynthesis, while the downregulated metabolic pathway was relevant to xenobiotic biodegradation, metabolism, and nucleotide metabolism. In summary, starter feeding before weaning significantly increased the dry matter intake and body weight of yak calves, changed the diversity and abundance of ruminal microbiota, and positively regulated the good development of ruminal morphology and function, providing an important basis for high-quality cultivation and the nutritional level of nutrition of yak calves in the Qinghai Tibet plateau. This study is based on the availability of 16S rRNA sequencing and LC-MS-based metabolomics in clarifying the function of starter feeding in the yak calves.

## Introduction

Yak calves are the foundation of the yak industry in the Qinghai-Tibetan Plateau, and the quality of yak calf rearing directly contributes to the performance of adults (Long et al., 2008). In the early stage of raising, the suckling period has a long-term impact on various biological functions (Baldwin et al., 2004; Soberon and Van Amburgh, 2013). Traditionally, yak calves are weaned naturally or artificially at 1.5–2 years of age under a wide range of conditions (Ding et al., 2013). Premature weaning of yaks is being attempted to improve ruminal growth and function (Mohr et al., 2002). However, under natural grazing conditions, there is a high mortality rate of calves due to the lack of nutrition and poor environmental conditions in the Qinghai-Tibetan Plateau (Liu et al., 2018). Taking this into account, Khan et al. (2016) proposed that a mixture of roughage and grain could be used for early weaning under the condition of house feeding. Diet, as one of the most important factors, affects the digestive systems, especially the structure and function of the ruminal microbiota, which can promote ruminal development (Berends et al., 2015; Latham et al., 2018; Ogunade et al., 2019). Coincidentally, a supplement of alfalfa stimulated the ruminal development consistent with the changes in ruminal microbiota and animal performance before and after weaning (Yang et al., 2018). Meanwhile, starter feeding decreased mRNA expression of cytokines, namely, TNF-α and IFN-γ in the colonic tissue and also in the digestive tract (Liu et al., 2017).

The digestive tract mainly develops in the early growth stage of calves, which effectively influences their long-term performance (Davis Rincker et al., 2011). As the fermentation pot of the ruminant animal, rumen fermentation produces volatile fatty acids (VFAs) that can directly stimulate the proliferation and development of the ruminal epithelium (Górka et al., 2018). Besides, the ruminal microbial composition is an effective way to resist external stimulation and maintain ruminal environmental stability (Shen et al., 2017). Therefore, the ruminal microbiota plays a central role in the efficiency of digestion in ruminants (Morgavi et al., 2013).

Although it had already been proved in dairy calves that alfalfa hay could promote ruminal epithelial and muscular development (Yang et al., 2015), but the inferior quality of alfalfa hay to be fed to calves could not provide efficient ruminal VFA required for ruminal papilla development (Mirzaei et al., 2015; Hosseini et al., 2016). Fortunately, the previous study found that the supplement of milk replacer and starter feeding can relieve the stress of weaning and raise the level of ruminal development (Sweeney et al., 2010). Therefore, in this study, 16S rRNA sequencing and metabolomics technology were used to investigate starter feeding based on alfalfa hay for weaning yak calves, providing an important reference for the research of milk replacer breeding technology after weaning in yak calves.

In recent years, next-generation high-throughput sequencing (*via* 16S rRNA sequencing) has been used to assess the influences of dietary on the ruminal microbial community (Pinloche et al., 2013; Kim et al., 2014). The application of metabolomics analysis provides an opportunity to measure large numbers of small molecule metabolites in cells, tissues, and biofluids (Goldansaz et al., 2017). Recent studies have applied metabolomics to predict feed efficiency and residual feed intake (Karisa et al., 2014; Artegoitia et al., 2017), examine disease conditions (Hailemariam et al., 2014), evaluate dietary responses to different feeds (Saleem et al., 2012), and assess milk quality of ruminants (Abarghuei et al., 2014). Nevertheless, variations in the types of metabolites produced as a result of starter feeding in yak calves have not been completely described. Therefore, a better comprehension of the relationship between starter feeding and ruminal factors (i.e., fermentation, morphology, microbiota community, and their metabolites) in yak calves will facilitate more accurate estimations of the starter supplement and demand under current feeding patterns.

## Materials and Methods

### Ethical Approval Statement

All yak calves and experimental protocols in this study were conducted following the recommendations of the Administration of Affairs Concerning Experimental Animals (Ministry of Science and Technology, China, revised in 2004).

### Animals and Experimental Design

All the yak calves were maternally nursed and grazed in the Datong Yak Breeding Farm of Qinghai Province before the trail. The experiment was conducted from July to November 2020, with 20 male yak calves at the age of 30 days [body weight (BW) of 33.67 ± 3.52 kg, mean ± standard deviation (SD)] with similar body conditions randomly recruited and assigned into two groups, with ten calves per group, nursed at the Haibei Tibetan Autonomous Prefecture Plateau Ecological Animal Husbandry Science Park Management Committee. All the calves were supplied with the same milk replacer (Beijing Precision Animal Nutrition Research Center, Q/HDJZA0007-2019); while the control (RA) group received only alfalfa hay, the treatment (RAS) group was fed with alfalfa hay and starter (Beijing Precision Animal Nutrition Research Center). The ten calves in each group were individually fed in ten different pens. We fed all the yak calves twice a day, at 08:00 and 16:30 with 100–700 g of milk replacer powder dissolved in 42°C water five times. The supplementation of milk replacer increased along with the increase of body weight, approximately 80 g per week until 3 months of age, and then gradually decreased by the same rate until the end of the study. Freshwater was supplied freely to the yak calves.

In brief, during the experimental period, the alfalfa hay and starter offered were adjusted daily to ensure at least 10% orts, while daily feed supplied was recorded at 3-day intervals, and the orts were gathered as well and then pooled and weighed at 3-day intervals for the calculation of the averaged dry matter intake (DMI) over 3 days until the average daily DMI achieved 1 kg each of the yak calves. At the end of the experiment, this resulted in the numbers of the feed intakes for each calf, and the means of those intakes were used as individual replicates for the statistical analysis of the difference in feed intake between the two treatments.

Samples of the starter feed, alfalfa hay, and milk replacer were measured (AOAC International, 2000) for dry matter (oven method 930.15), sugar (colorimetric method), starch (α-amylase method), crude protein (Kjeldahl method 988.05), ether extract (alkaline treatment with Röse-Gottlieb method 932.06 for milk replacer; diethyl ether extraction method 2003.05 for starter and alfalfa hay), NDF with ash without sodium sulfite or α-amylase, ADF with ash, calcium (Ca), and phosphorus (P) (dry ashing, acid digestion, and analysis by inductively coupled plasma, method 985.01), and the nutrient compositions of the milk replacer, alfalfa hay, and starter are given in Table 1.

**TABLE 1:** Nutrient composition of the milk replacer, alfalfa hay, starter used in the present study.

Items (% of dry matter)	Milk replacer^1^	Alfalfa hay	Starter feed
Dry matter (% as fed)	95.00	93.70	87.80
Sugar	−	−	6.50
Starch	−	−	40.50
Crude protein	26.24	12.51	20.01
Ether extract	27.79	0.89	4.70
Neutral detergent fiber	−	56.45	10.90
Acid detergent fiber	−	40.40	4.10
Calcium	2.50	0.99	0.79
Phosphorus	1.40	0.16	0.46
Lysine	2.20	0.84	1.05
Methionine	1.00	0.16	0.34

*^1^The milk replacer was stored as a powder and contained whole milk powder, whey powder, protein concentrate, vitamin A, vitamin D3, vitamin E, nicotinic acid, pantothenic acid, lysine, methionine, threonine, sodium chloride, copper, zinc manganese, and iron.*

### Sample Collection

When the average daily DMI achieved 1 kg each of the yak calves, yak calves were fasted for 24 h, and then, the body weight, height, length, and the chest girth of all the yak calves were recorded. Five yak calves were selected randomly from each group, killed by exsanguination, and then dissected at once.

The rumen was separated, and the content within the rumen was collected for sampling. We collected 5 ml of homogenized ruminal content samples in triplicates from the ventral sac of the rumen and stored them at -80°C for microbial DNA extraction and untargeted metabolomics. Ruminal fluid samples were collected from individual yak calves and strained through 4 layers of sterile cheesecloth, and the pH was measured immediately using a portable pH meter (HI 9024C; HANNA Instruments, Woonsocket, RI, United States). Meanwhile, another 5 ml of the ruminal fluid was collected and stored at −20°C for VFA and NH_3_-N analyses. Specifically, a solute with metaphosphoric acid and crotonic acid was added to 2 ml of these 5 ml ruminal fluid samples before further analyses of the VFA concentrations in gas chromatography (GC-14B, Shimadzu, Japan) (Wang et al., 2017).

Subsequently, three segments of the tissue sample (2 × 2 cm) from the ventral sac of the rumen were collected, immediately washed with saline solution, then fixed in 10% buffered formalin, and stored at 4°C until papilla length and width, and the thickness of ruminal base was measured (Ishii et al., 2005). All the tissue samples were taken from the same location in each animal. Additionally, all the other collected samples were first stored in liquid nitrogen for 24 h, unless noted otherwise, and then stored at −80°C before analyses.

### Determination of the Ruminal Morphology

By using the routine method of the wax section, the development of rumen was studied. After fixing in 10% buffered formalin for 24 h, the ventral sac of the ruminal tissue samples was gradually dehydrated at different concentrations (60, 70, 80, 90, and 100%) of ethanol and cleaned. Then, the ruminal samples were trimmed into small pieces and inserted into cassettes, which were embedded in liquid paraffin. Notably, 5-μm paraffin sections were sliced using the microtome and stained with hematoxylin-eosin. Using the phase-contrast microscope (Nikon NiE200, Tokyo, Japan) the papillae length and width of the ruminal tissue and the thickness of the ruminal base were measured (Wu et al., 2018).

### Determination of Volatile Fatty Acid and NH_3_-N Concentrations in Ruminal Fluid

The ruminal fluid samples were centrifuged at 13,000 × *g* for 10 min at 4°C before the VFA and NH_3_-N concentration measurement. Using the Agilent 6850 gas chromatograph (Agilent Technologies Inc., Santa Clara, CA, United States) equipped with a polar capillary column (HP-FFAP, 30 m × 0.25 mm × 0.25 μm) and a flame ionization detector to analyze the supernatant ruminal fluid samples is previously described (Xue et al., 2017). The NH_3_-N concentration in each supernatant sample was measured using a continuous-flow analyzer (SKALAR San, Skalar Co., Breda, Netherlands).

### Microbial DNA Extraction, 16S rRNA Gene Amplification of the V3 + V4, and Bioinformatics Analysis

Total genome DNA from the 10 ruminal content samples of yak calves from two different treatments was extracted using the cetyltrimethylammonium bromide (CTAB) method in accordance with Henderson et al. (2013). Meanwhile, DNA extraction was assessed through the QIAamp DNA Stool Mini Kit (Qiagen, Dusseldorf, Germany). DNA concentration was monitored on 1% agarose gels. The purity was assessed from the 260: 280 nm ratio (>1.8) using a NanoDrop ND2000 spectrophotometer (Thermo Scientific, Waltham, MA, United States), and the DNA was stored at −80°C until it was used in sequencing analysis.

16S rRNA genes of 16S V3-V4 regions were amplified using specific primer set 515F (5′-GTGCCAGCMGCCGCGG-3′) and 806R (5′-GGACTACNNGGGTATCTAAT-3′) with barcodes (Yu et al., 2005; Sundberg et al., 2013). All PCR reactions were carried out with 15 μl of Phusion^®^ High-Fidelity PCR Master Mix (New England Biolabs, Beijing, China), 2 μM of forward and reverse primers, and approximately 10 ng template DNA. Thermal cycling consisted of initial denaturation at 98°C for 1 min, followed by 30 cycles of denaturation at 98°C for 10 s, annealing at 50°C for 30 s, and elongation at 72°C for 30 s, and finally, 5 min at 72°C. PCR product quantification and qualification: the same volume of 1 × loading buffer (contained SYB green) was mixed with PCR products and electrophoresis on 2% agarose gel was performed for detection. PCR products were mixed in equidensity ratios. Then, the mixture of PCR products was purified using the Qiagen Gel Extraction Kit (Qiagen, Dusseldorf, Germany). Sequencing libraries were generated using the TruSeq^®^ DNA PCR-Free Sample Preparation Kit (Illumina, San Diego, CA, United States) following the manufacturer’s recommendations and adding index codes. The library quality was assessed using the Qubit@ 2.0 Fluorometer (Thermo Scientific, Waltham, MA, United States) and Agilent Bioanalyzer 2100 system. Finally, the library was sequenced on an Illumina NovaSeq platform, and 250 bp paired-end reads were generated.

Quality filtering on the raw tags was performed under specific filtering conditions to obtain the high-quality clean tags (Bokulich et al., 2013), according to the QIIME 2 (Bolyen et al., 2019). The tags were compared with the reference database (Silva database) using the UCHIME algorithm (UCHIME) (Edgar et al., 2011) to detect chimera sequences, then the chimera sequences were removed (Haas et al., 2011), and effective tags were finally obtained.

Sequence analyses were performed using Uparse software (Uparse version 7.0.1001) (Edgar, 2013). Sequences with ≥97% similarity were assigned to the same OTUs. A representative sequence for each OTU was screened for further annotation. For each representative sequence, the Silva database (Edgar et al., 2011) was used based on the Mothur algorithm to annotate taxonomic information. To study the phylogenetic relationship of different OTUs and the difference of the dominant species in different groups, multiple sequence alignments were conducted using the MUSCLE software (Version 3.8.31) (Edgar, 2004). OTU abundance information was normalized using a standard sequence number corresponding to the sample with the least sequences. Subsequent analyses of alpha diversity and beta diversity were all performed based on this output normalized data.

The taxon abundance for each sample was determined according to phylum, class, order, family, and genus. The microbiota were compared for beta diversity using the distance matrices generated from weighted UniFrac analysis, principal coordinated analysis (PCoA), and analysis of similarities (ANOMIS). The *P-*value was set as <0.05, and the threshold of the linear discriminant analysis (LDA) score was set at a default value of 2.0.

### Untargeted Metabolomics

The ruminal content samples (1 ml) were freeze-dried and resuspended with prechilled 80% methanol and 0.1% formic acid using a good vortex. Then, the samples were incubated on ice for 5 min and centrifuged at 15,000 *g*, 4°C for 15 min. Some supernatant was diluted to the final concentration containing 53% methanol by LC-MS grade water. The samples were subsequently transferred to a fresh Eppendorf tube and then centrifuged at 15,000 *g*, 4°C for 15 min. Finally, the supernatant was injected into the LC-MS system analysis.

Discoverer 3.1 (CD3.1, ThermoFisher, Waltham, MA, United States) was used to perform peak alignment, peak picking, and quantitation for each metabolite. Later, peak intensities were normalized to the total spectral intensity. The normalized data were used to predict the molecular formula based on additive ions, molecular ion peaks, and fragment ions. Then, peaks were matched using the mzClou, mzVault, and MassList database to obtain accurate qualitative and relative quantitative results.

Statistical analyses were performed using the statistical software R (R version R-3.4.3), Python (Python 2.7.6 version), and CentOS (CentOS release 6.6). When data were not normally distributed, normal transformations were attempted using area normalization.

These metabolites were annotated using the Kyoto Encyclopedia of Genes and Genomes (KEGG) database. Principal component analysis (PCA) was performed at metaX (Wen et al., 2017) (a flexible and comprehensive software for processing metabolomics data). We applied univariate analysis (*t*-test) to calculate the statistical significance (*P*-value). The metabolites with VIP > 1, *P*-value < 0.05, and fold change (FC) ≥ 2 or FC ≤ 0.5 were considered to be differential metabolites. Volcano plots were used to filter metabolites of interest-based on log_2_(FC) and −log_10_(*P*-value) of metabolites using ggplot2 in R language.

The KEGG pathway enrichment of differential metabolites was performed, the ratio was satisfied by x/n > y/N, and the metabolic pathway was considered an enrichment, while when the *P*-value of the metabolic pathway < 0.05, the metabolic pathway was considered a statistically significant enrichment.

### Statistical Analysis

In this study, all data were tested and presented as a normal distribution. At the beginning of the experiment, the initial body weight, height, length, and chest girth were tested using the *t*-test, no significant differences were identified, and the initial indices were almost constant. Therefore, for other indices except for the DMI data, analysis was performed using the one-way ANOVA procedure using SPSS 22.0 software (SPSS, Inc., Chicago, IL, United States) with replicates as experiment units, and a value of *P* < 0.05 was regarded as statistically significant. The differences in DMI between two groups of yak calves were further analyzed using the following model: Yij = μ + Di + Tj + εij + DT, using the MIXED procedure of SAS (SAS Institute Inc., Cary, NC, United States, 2007), which considered the significant differences of these indices induced by the time effect in the same treatment. Yij is the response variable, in particular, μ is the overall mean, Di is the fixed effect of treatment (*i* = maternal grazing or barn feeding), Tj is the fixed effect of time (10 days as a unit) of the experiment, DT is the interaction of dietary and time, and εij is the residual error. If a significant diet and time effect were observed, the significance between the treatment and time differences was separately identified using the Tukey’s test multiple comparison test. All data are expressed as the means with the standard error.

The statistical evaluation of 16S rRNA sequencing and the untargeted metabolomics results were analyzed using the bioinformatics methods described above. Spearman’s correlation test was used to examine relationships between the RA group and the RAS group. The correlation coefficient rho between the relative abundance of differential metabolites and the total abundance of differential bacteria was calculated using the Spearman statistical method (rho ≥ 0, *P* ≤ 0.05).

## Results

### The Effect of Starter Feeding on Growth Performance, Dry Matter Intake, and Development of the Rumen

Significant differences in daily DMI were found between the two groups of the yak calves over the whole experimental period (*P* = 0.033), where the higher intake was found for yak calves in the RAS group. Compared with the RA group, the RAS group had a significantly greater body weight (*P* < 0.001), height (*P* = 0.008), and chest girth (*P* < 0.001) (Table 2). Meanwhile, a significant increase in organ development was found in the RAS group, which included the weight of the rumen (*P* = 0.036) (Table 2).

**TABLE 2:** Effects of the starter feeding supplementation in the preweaning period on the growth performance and development of rumen in yak calves.

		Treatment^1^		SEM	*P*-value
Items		RA	RAS		
Growth performance	Body weight (kg)	72.07 ± 2.47	80.58 ± 1.26	1.38	< 0.001
	Body length (cm)	90.80 ± 3.63	93.80 ± 1.92	1.34	0.141
	Body height (cm)	76.33 ± 2.08	81.25 ± 0.96	1.20	0.008
	Chest girth (cm)	104.80 ± 2.68	116.40 ± 2.61	2.22	< 0.001
	DMI (g)	608.36 ± 60.68	678.71 ± 6.53	17.04	0.033
Rumen weight	Rumen (g)	1.07 ± 0.07	1.17 ± 0.04	0.23	0.036
Rumen index, expressed as kg of organ/kg of BW	Rumen (×10^–2^)	1.47 ± 0.08	1.56 ± 0.13	0.17	0.221

*^1^The alfalfa (RA) was fed with the milk replacer and alfalfa hay, the alfalfa hay and starter (RAS) group was fed with milk replacer, alfalfa hay and the starter.*

### Development of Ruminal Morphology and Ruminal Fermentation Profiles

Over the whole experimental period, when compared with the RA group, the RAS group had significantly greater papilla length (*P* = 0.049). The RAS group could significantly increase the ruminal fluid NH_3_-N concentration compared with the RA group (*P* = 0.001). Besides, the RA group had a significantly higher acetate concentration than the yak calves in the RAS group (*P* = 0.034), while the valerate concentration was significantly greater in the RAS group (*P* = 0.021) (Table 3).

**TABLE 3:** Effects of starter feeding on the ruminal fermentation parameters and ruminal epithelium development in yak calves.

		Treatment		SEM	*P*-value
Items		RA	RAS		
Ruminal epithelium	Papilla width (μm)	952.69 ± 105.45	824.07 ± 191.11	25.15	0.283
	Papilla length (μm)	569.82 ± 100.37	748.72 ± 128.53	33.29	0.049
	Muscle thickness (μm)	1915.07 ± 439.49	2031.12 ± 643.69	105.68	0.757
Ruminal fermentation characteristics	pH	7.35 ± 0.12	7.35 ± 0.116	0.03	0.978
	Ammonia nitrogen, NH_3_-N (mg/dL)	2.46 ± 0.28	3.29 ± 0.23	0.16	0.001
	Total VFA (mmol/L)	57.40 ± 5.27	50.23 ± 5.25	2.07	0.081
	Acetate (mmol/L)	35.78 ± 3.51	29.60 ± 1.05	0.23	0.034
	Propionate (mmol/L)	9.39 ± 1.29	8.28 ± 1.07	0.44	0.232
	Butyrate (mmol/L)	5.41 ± 1.55	4.62 ± 1.18	0.43	0.389
	Isobutyrate (mmol/L)	1.53 ± 0.084	1.54 ± 0.25	0.06	0.936
	Valerate (mmol/L)	3.58 ± 0.43	4.23 ± 0.05	0.15	0.021
	Isovalerate (mmol/L)	1.20 ± 0.14	1.27 ± 0.17	0.05	0.592

### Ruminal Microbiota

**FIGURE 1:**
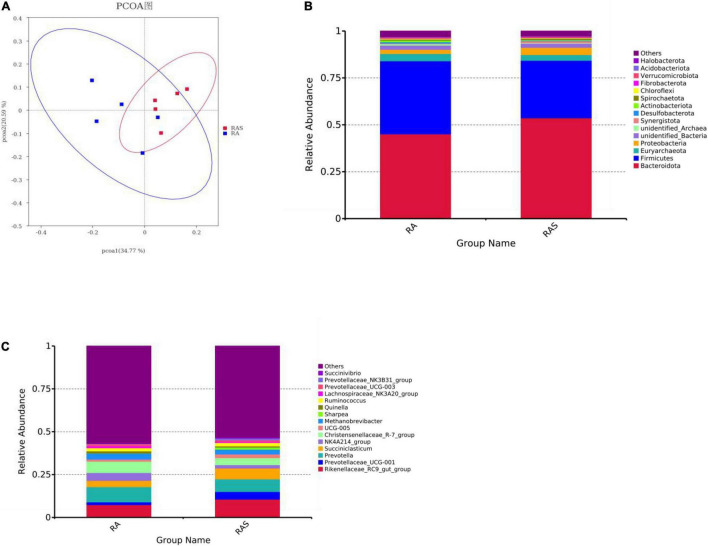
**(A)** Diversity analyses based on the ruminal microbiota; phylum **(B)** and genus level **(C)** composition of the ruminal bacteria (top 15).

According to further analysis of ruminal microbiota, we found significantly decreased the OTUs in the RAS group (Table 4, *P* = 0.046), whereas the two groups showed no significant difference in Chao 1 value, Shannon indices, and Simpson index (Table 4, *P* = 0.468). Moreover, beta diversity analyses showed that the compositions of the gastrointestinal prokaryotic community of the yak calves in different feeding groups were different in the rumen (Figure 1A).

**TABLE 4:** Effects of the starter feeding on richness and diversity index of ruminal microbiota in yak calves.

Items	Treatment		SEM	*P*-value
	RA	RAS		
OTUs	1457.25 ± 94.49	1265.00 ± 120.69	50.78	0.046
Good’s coverage	0.99 ± 0.001	0.99 ± 0.001	0.01	0.557
ACE value	1619.81 ± 162.03	1404.46 ± 132.99	63.33	0.086
Chao 1 value	1596.47 ± 143.09	1385.30 ± 136.44	60.72	0.077
Shannon indices	7.50 ± 0.29	7.18 ± 0.89	0.21	0.468
Simpson indices	0.97 ± 0.01	0.96 ± 0.03	0.01	0.468

Rumen whose phylum-level relative abundance was in the top 15 of all the microbiota communities is shown in Figure 1B. The genus of the top 15 is given in Figure 1C. Differential ruminal microbiota are identified between two different feeding groups. Among these, *Firmicutes* (*P* < 0.001), *Desulfobacterota* (*P* = 0.002), and *Actinobacteriota* (*P* = 0.002) were significantly greater in the RA group, while *Bacteroidota* (*P* = 0.001), *Proteobacteria* (*P* = 0.011), *Chloroflexi* (*P* = 0.004), *Synergistota* (*P* = 0.005), and *Spirochaetota* (*P* = 0.017) were significantly higher in the RAS group (Table 5). At the genus level, *Prevotella* (*P* = 0.018), *Christensenellaceae_R-7_group* (*P* = 0.013), and *NK4A214_group* (*P* = 0.018) were significantly greater in the RA group. *Rikenellaceae_RC9_gut_group* (*P* = 0.004) and *Ruminococcus* (*P* = 0.041) were identified significantly greater genera in the RAS group. Furthermore, similar results that contained similar but less differential bacteria were also identified using the Mann–Whitney *U* test (Table 5).

**TABLE 5:** Ruminal microbiota community difference between the two groups by using the Mann–Whitney *U* test.

Items	Groups		SEM	*P*-value
	RA	RAS		
**Phylum**				
*Bacteroidota*	38.38 ± 4.96	53.61 ± 1.41	2.963	0.001
*Firmicutes*	43.98 ± 2.77	30.70 ± 2.41	2.567	< 0.001
*Euryarchaeota*	3.16 ± 1.16	2.20 ± 0.92	0.387	0.240
*Proteobacteria*	2.68 ± 0.38	3.87 ± 0.59	0.263	0.011
*Verrucomicrobiota*	0.38 ± 0.13	0.43 ± 0.15	0.046	0.577
*Chloroflexi*	0.13 ± 0.04	0.32 ± 0.07	0.040	0.004
*Synergistota*	0.27 ± 0.05	0.90 ± 0.27	0.140	0.005
*Desulfobacterota*	0.68 ± 0.11	0.34 ± 0.08	0.069	0.002
*Actinobacteriota*	1.06 ± 0.28	0.28 ± 0.02	0.169	0.002
*Spirochaetota*	0.46 ± 0.19	1.00 ± 0.22	0.130	0.017
**Genus**				
*Prevotella*	11.04 ± 0.10	8.10 ± 1.43	0.707	0.018
*Methanobrevibacter*	2.67 ± 0.74	1.20 ± 0.85	0.290	0.274
*Quinella*	0.56 ± 0.08	0.56 ± 0.35	0.080	0.993
*Rikenellaceae_RC9_gut_group*	6.73 ± 1.74	12.33 ± 0.79	1.240	0.004
*Prevotellaceae_UCG-001*	1.56 ± 0.31	1.39 ± 0.54	0.164	0.659
*Succiniclasticum*	3.66 ± 0.92	4.57 ± 0.50	0.337	0.203
*NK4A214_group*	2.72 ± 0.71	1.43 ± 0.25	0.311	0.018
*Christensenellaceae_R-7_group*	6.60 ± 1.11	4.07 ± 1.40	0.565	0.013
*UCG-005*	1.37 ± 0.46	1.08 ± 0.11	0.139	0.351
*Ruminococcus*	1.59 ± 0.41	3.93 ± 0.23	0.660	0.041

### Microbiological Metabolism of the Rumen

**FIGURE 2:**
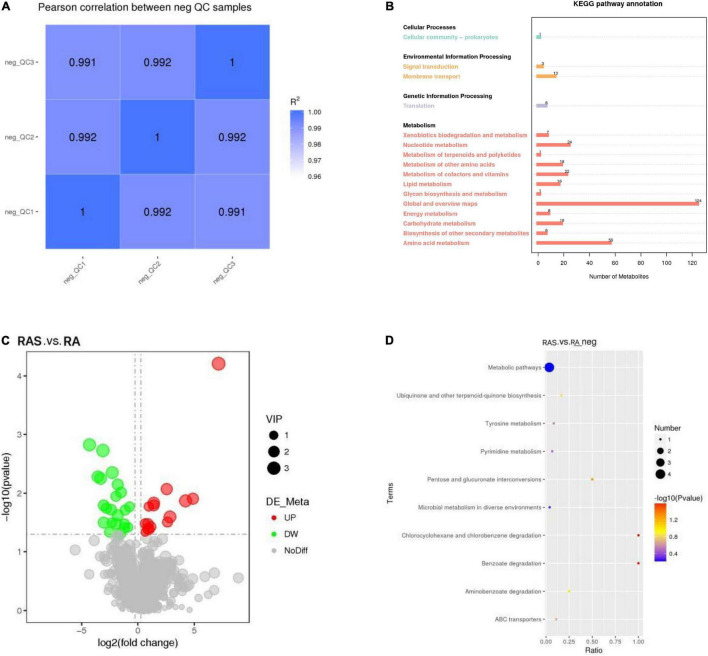
**(A)** QC samples correlation analysis; **(B)** The Kyoto Encyclopedia of Genes and Genomes (KEGG) pathway annotation; **(C)** The volcano plot of difference metabolites; **(D)** enrichment of differential metabolites in KEGG pathways. Rich factor, ratio of the proportion of differential metabolites to the proportion of all metabolites in the pathway; the size of the dots in the graph represents the number of distinct metabolites enriched in the corresponding pathways.

Based on the relative quantitative value of the metabolites, the Spearman correlation coefficient between the QC samples is calculated (Figure 2A). The correlation between the QC samples was closer to 1 (*R*^2^ < 1), indicating that the test process was stable and the quality of data high (Rao et al., 2016).

In contrast, metabolism mainly participated in amino acid metabolism and nucleotide metabolism in ruminal content (Figure 2B). We detected the metabolites in the rumen by metabolomics. In our research, the relative concentrations of 37 of the total 1,057 identified metabolites were altered by starter feeding (Figure 2C), of which 15 metabolites were upregulated and 22 metabolites were downregulated in yak calves in the RAS group compared with those in the RA group (Table 6).

**TABLE 6:** Differential metabolites in the rumen of yak calves in the RA and RAS groups.

Metabolites	mzmed	rtmed	log2FC	*P*-value	VIP	Regulated
Saccharin	181.992	6.042	7.160	0.000	3.387	Up
6-Keto-prostaglandin f1alpha	369.228	12.182	2.556	0.009	2.381	Up
delta-Tocopherol	401.343	16.697	4.883	0.012	2.348	Up
Pyridoxine *O*-Glucoside	330.120	2.068	4.228	0.014	2.896	Up
2′-*O*-Methyluridine	257.079	5.528	1.411	0.015	2.638	Up
15(S)-HpETE	353.234	12.929	1.405	0.016	2.137	Up
FAHFA (16:1/18:3)	529.424	14.506	0.964	0.017	1.374	Up
Isopentenyladenine	202.110	10.799	2.845	0.025	2.731	Up
*N*1-isopropyl-2-(1H-2-pyrrolylcarbonyl)-1-hydrazinecarboxamide	419.212	9.289	2.629	0.031	1.717	Up
*N*7-Methylguanosine	298.115	1.361	0.638	0.033	1.566	Up
Pepstatin	684.455	15.539	0.884	0.033	2.031	Up
(+/-)8(9)-DiHET	337.239	13.317	1.122	0.038	2.039	Up
Xylitol	151.061	1.426	0.862	0.041	1.692	Up
(+/-)9,10-dihydroxy-12Z-octadecenoic acid	313.239	12.924	0.962	0.041	2.172	Up
Cinnamoylglycine	204.067	8.755	0.639	0.046	1.025	Up
3,5-Dihydroxybenzoic acid	153.020	7.550	–4.292	0.001	2.951	Down
(3R)-8-hydroxy-3-(4-methoxyphenyl)-3,4-dihydro-1H-2-benzopyran-1-one	269.083	9.621	–3.107	0.002	3.140	Down
2-Hydroxyhippuric acid	194.046	7.134	–2.277	0.004	2.801	Down
FAHFA (2:0/23:0)	411.349	16.143	–3.557	0.005	2.496	Down
Hydroquinone	109.030	7.551	–3.315	0.006	2.674	Down
D-Glucosyl-beta-1,1-*N*-palmitoyl-D-erythro-sphingosine	698.560	16.209	–1.790	0.007	2.303	Down
3-(methylsulfanyl)-5H-[1,2,4]triazino[5,6-b]indole	215.039	10.572	–1.497	0.010	2.102	Down
PC (15:1/18:2)	800.546	16.225	–1.955	0.011	1.749	Down
Isorhapontigenin	257.082	10.283	–3.028	0.016	2.153	Down
FAHFA (2:0/18:1)	357.265	13.102	–0.751	0.017	1.655	Down
1-(2,4-dihydroxyphenyl)-2-(3,5-dimethoxyphenyl)propan-1-one	301.108	7.583	–2.793	0.018	1.856	Down
2-Furoylglycine	168.031	5.992	–2.379	0.019	2.147	Down
PE (16:0/16:0)	690.509	14.288	–1.134	0.019	1.724	Down
D-Glucuronic acid	193.036	12.638	–1.792	0.024	2.331	Down
PG (20:0/20:4)	825.567	15.518	–3.036	0.032	2.263	Down
PG (20:0/20:3)	827.584	16.042	–2.299	0.032	1.490	Down
13,14-dihydro Prostaglandin F1α	393.243	14.550	–1.955	0.033	2.025	Down
PG (18:1/22:4)	823.552	15.392	–1.183	0.033	1.623	Down
3-methyl-4-[2-(2-methylphenyl)hydrazono]-4,5-dihydro-1H-pyrazol-5-one	215.093	1.915	–0.860	0.038	1.362	Down
UMP	323.029	1.460	–1.210	0.039	2.018	Down
*N*1-[4-(cyanomethyl)phenyl]-4-chlorobenzamide	269.046	10.375	–2.514	0.046	1.759	Down
Catechin	289.072	7.111	–1.567	0.049	2.133	Down

*FC, fold change; mzmed, mass-to-charge ratio of metabolites; rtmed, retention time of metabolites; VIP, variable importance in the projection.*

*Regulated “up” represents a higher abundance in calves in the RAS group, “down” represent a higher abundance in calves in the RA group.*

As shown in Figure 2D, UMP, hydroquinone, xylitol, and delta-tocopherol were enriched in the KEGG pathway “metabolic pathways.” Hydroquinone was enriched in the KEGG pathway “Microbial metabolism in diverse environments,” “Tyrosine metabolism,” “Aminobenzoate degradation,” “Benzoate degradation,” and “Chlorocyclohexane and chlorobenzene degradation.” Xylitol was enriched in the KEGG pathway “ABC transporters” and “Pentose and glucuronate interconversions.” UMP and delta-tocopherol may be related to “Pyrimidine metabolism” and “Ubiquinone and other terpenoid-quinone biosynthesis,” respectively.

### Correlation Between Ruminal Microbiota and Fermentation Profiles

**FIGURE 3:**
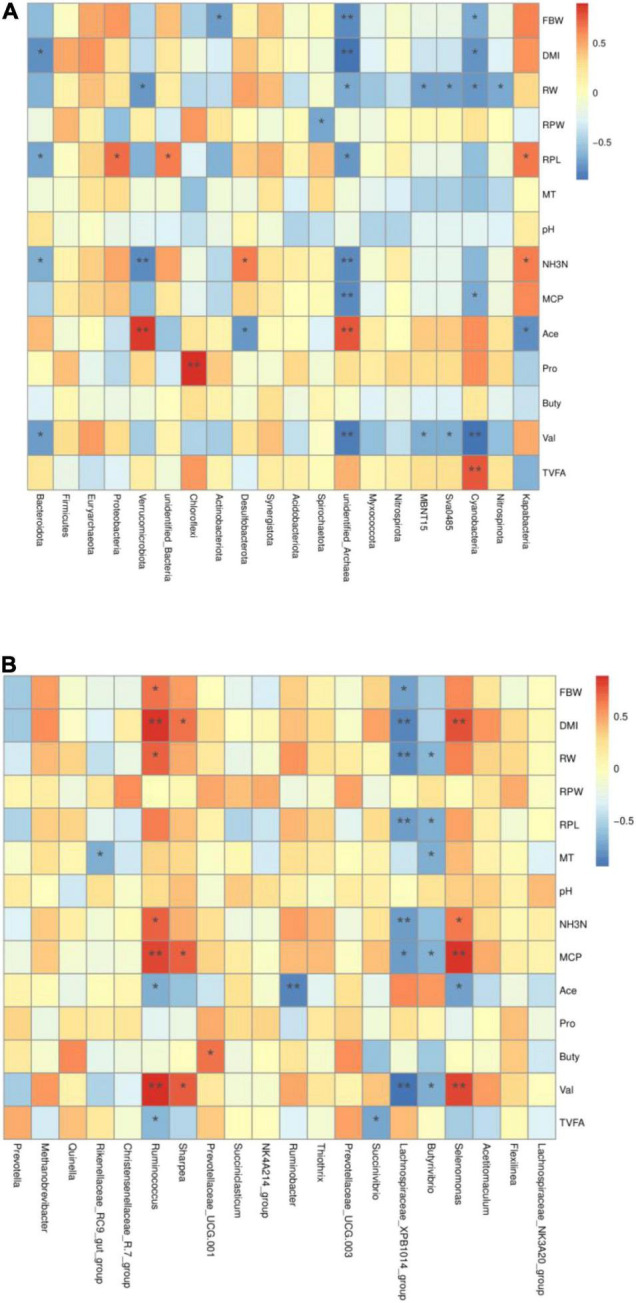
Heatmap of the correlation between microbiota [**(A)** phylum and **(B)** genus] and fermentation parameters in the rumen of yak calves. RBW, finally body weight; DMI, dry matter intake; RW, ruminal weight; RPW, ruminal papilla width; RPL, ruminal papilla length; MT, muscle thickness; NH3N, NH_3_-N; MCP, microbiota crude protein; Ace, acetate; Pro, propionate; But, butyrate; Ibu, isobutyrate; Val, valerate; Iva, isovalerate.

There is an interaction between ruminal microbiota and growth performance of yak calves and ruminal fermentation profiles, which affects the feed efficiency and tissue development of ruminants. The correlation between them is illustrated in Figure 3.

At the phylum level (Figure 3A), finally, body weight of yak calves was significantly positively correlated with *Actinobacteriota*, *unidentified-Archaea*, and *Cyanobacteria*. The DMI of yak calves was significantly negatively correlated with *Bacteroidota*, *unidentified-Archaea*, and *Cyanobacteria*. The ruminal weight of yak calves was significantly negatively correlated with Verrucomicrobiota, *unidentified-Archaea*, *MBNT15*, *Sva0485*, *Cyanobacteria*, and *Nitrospinota*. The width of ruminal papilla was significantly negatively correlated with *Spirochaetota*. The length of ruminal papilla was significantly positively correlated with *Proteobacteria*, *unidentified-Bacteria*, and *Kapabacteria* and significantly negatively correlated with *Bacteroidota* and *unidentified-Archaea*. The NH_3_-N concentration in the ruminal fluid was significantly negatively correlated with the *Bacteroidota*, *unidentified-Archaea*, and *Verrucomicrobiota* and was significantly positively correlated with *Desulfobacterota* and *Kapabacteria*. The MCP concentration in the ruminal fluid was significantly negatively correlated with the relative abundance of *unidentified-Archaea* and *Cyanobacteria*. Acetate concentration was significantly positively correlated with *Verrucomicrobiota* and *unidentified-Archaea* and significantly negatively correlated with Kapabacteria. Propionate concentration was significantly positively correlated with *Chloroflexi*. The concentration of valerate was significantly negatively correlated with *Bacteroidota*, *unidentified-Archaea*, MBNT15, Sva0485, and *Cyanobacteria*. TVFA concentration was significantly positively correlated with *Cyanobacteria*.

At the genus level (Figure 3B), there were significant positive correlations between the final body weight, DMI, ruminal weight, NH_3_-N, MCP concentration, and valerate concentration and the relative abundance of *Ruminococcus*, significant negative correlations between acetate and TVFA concentration and the relative abundance of *Ruminococcus*. MCP and valerate concentrations were significantly positively correlated with *Sharpea*. Butyrate concentration was significantly positively correlated with the *Prevotaceae-UCG-001*. The concentration of acetate was significantly negatively correlated with *Ruminobacter*. The final body weight, DMI, ruminal weight, ruminal papilla length, NH_3_-N, and MCP concentration and valerate concentration of yak calves were significantly negatively correlated with *Lachnospiraceae-XPB1014-group*.

### Correlation Between Ruminal Microbiota and Metabolites

**FIGURE 4:**
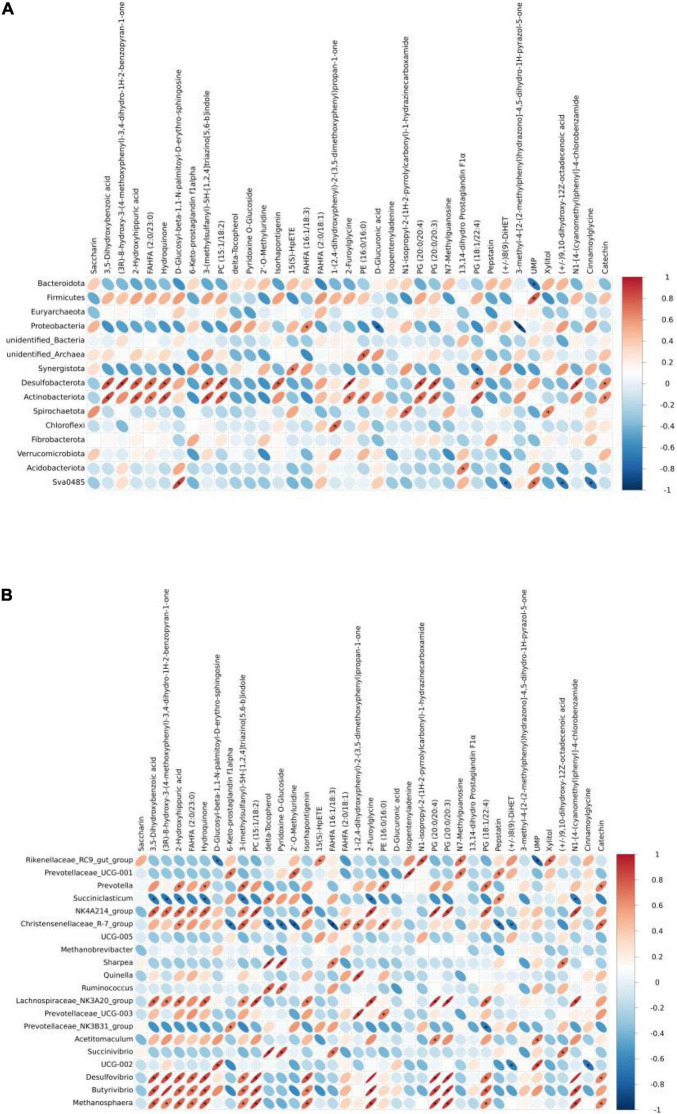
Heatmap of the correlation between ruminal microbiota [**(A)** phylum and **(B)** genus] and metabolites of yak calves.

In this study, the correlation analysis results of metabolites and 16S rRNA are shown. As shown in Figure 4A, a total of 44 correlations significantly differ at the phylum level, with 36 significantly positive correlations and 8 significantly negative correlations. Hydroquinone was significantly positive with *Desulfobacterota* and *Actinobacteriota*. UPM was significantly positive with *Firmicutes* and *Sva0485*, while significantly negative with *Bacteroidota*. Xylitol was significantly positive with *Spirochaetota*.

There were 104 significantly positive and 19 significantly negative correlations between ruminal microbiota and their metabolites at the genus level (Figure 4B), with *Rikenellaceae_RC9_gut_group* significantly positive with UPM and significantly positive with xylitol. *Prevotell*a, *NK4A214_group*, *Lachnospiraceae_NK3A20_group*, *Desulfovibrio*, *Butyrivibrio*, and *Methanosphaera* were significantly positive with Hydroquinone, while *Succiniclasticum* was significantly negative with Hydroquinone. Delta-tocopherol was significantly positive with *Succiniclasticum*, *Sharpea*, *Ruminococcus*, and *UCG-002*, while it was significantly negative with *Christensenellaceae_R-7_group*. UMP was significantly positive with *Acetitomaculum* and *UCG-002*.

## Discussion

Due to the slow development of rumen, the growth performance of newborn calves was related to the digestion of wrinkles and the intestine. The ruminal epithelium is responsible for nutrient absorption and transportation in the rumen. This study shows that starter feeding with alfalfa hay significantly increased DMI, body weight, and chest girth in yak calves, which presumably stimulated both the development of ruminal epithelia and performance (Baldwin et al., 2004; Norouzian et al., 2011; Yang et al., 2015; Lin et al., 2019), in line with the results of the previous studies in calves’ and lambs’ early life (Saro et al., 2018; Cui et al., 2020). Starter feeding also significantly promoted ruminal development in yak calves. These improvements probably attributed to the improved dry matter intake and ruminal fermentation. With the growth of the age and the solid feed intake, the ruminal epithelium can influence the net use of digestion and utilization of diet, serving as a barrier to the ruminal epithelium’s contents (Sander et al., 1959; Anderson et al., 1987). Similar to the previous study, the starter feeding contributed to the ruminal papilla length, which is also consistent with Xie et al. (2013) research on Holstein calves.

Due to various in-nutrient supplementation, significant changes occurred in the ruminal fermentation, which represented the increased carbohydrate fermentation in the rumen (Raghuvansi et al., 2007; Xue et al., 2017; Yang et al., 2018). The starter feeding contains grains, thus providing easily fermentable carbohydrates for microbial fermentation. When supplied with a starter, the RAS group produced a bigger NH_3_-N concentration compared with the RA group, which fed with only alfalfa hay. NH_3_-N concentration in the rumen is in a dynamic balance, which can provide raw materials for generating the microbial protein (Firkins et al., 2007). Increased NH_3_-N concentration in RAS group yak calves, this is in line with this study (Agle et al., 2010). As suggested in previous studies (Stams et al., 1984; Poudel et al., 2018; Gao et al., 2019), the alfalfa hay supplementation could increase the ruminal acetate-producing bacteria, further influencing the ruminal acetate concentration. Likewise, an increased acetate concentration level in the RA group calves that fed with the alfalfa hay reflected increased fiber digestibility because the acetate is the primary product of cellulolytic bacteria (Lu et al., 2005). The significantly increased valerate concentration happened in the starter feeding group, which demonstrated that more nutrients could be provided to increase growth performance and organ development (Orskov, 2012).

Both dietary and additives could influence the abundance of ruminal microbiota (Abecia et al., 2014), while the abundance of the microbiota also affects the use of the host’s utilization of the diet (Carberry et al., 2014). *Bacteroidetes* and *Firmicutes* are the most dominant phylum in yak calves’ rumen, which are consistent with the previous studies on ruminants (Shen et al., 2017; Wu et al., 2021). The depressed ratio of *Firmicutes* vs. *Bacteroidetes* in the rumen resulted in increasing lignocellulose digestion (Mu et al., 2019). Similar to other studies (Fernando et al., 2010; Iqbal et al., 2017), the dominant phylum was identified in this study, including *Euryarchaeota* and *Proteobacteria*. Through the correlation analysis, *Proteobacteria* and *Synergistota* were significantly positive with the NH_3_-N concentration, and these three indicators were also significantly higher in the starter feeding group, which indicated that starter feeding can promote ruminal microbiota to produce available NH_3_-N and strengthen the absorption and utilization of nutrients. *Proteobacteria* have the function of degrading soluble carbohydrates; the crude protein and ether extract were much higher in the feeding of the RAS group, along with the significantly increased abundance of *Proteobacteria* in the RAS group. Therefore, we predicted that the abundance of *Proteobacteria* was positive with the crude protein and ether extract content in the dairy feed.

*Prevotella* was the dominant genus of abundance, which had the ability to degrade fiber sources (Emerson and Weimer, 2017). Members of the genus *Prevotella* in the ruminal microbial community have been reported to play an important role in the utilization of dietary nutrition (Latham et al., 2018). In this study, the abundance of *Prevotella* was significantly negative with isobutyrate, and the abundance of *Prevotella* was significantly increased in the RA group, due to the higher neutral detergent fiber and acid detergent fiber level in the alfalfa hay. Part strains of *Butyrivibrio* degraded cellulose in the rumen, which also shows that the abundance of *Butyrivibrio* increased in the RA group. The abundance of *Rikenellaceae_RC9_gut_group* was significantly enriched in the RAS group and positive with the NH_3_-N and valerate concentration, which was consistent with the result of rumen fermentation. The relative abundance of *UCG-002* was negative with the propionate, butyrate, valerate, and isovalerate concentration, which could be resistant to the growth performance when propionate was absorbed and converted to glucose, amino acids, and lipids (Orskov, 2012). Very few studies referred to the significant valerate concentration decrease in the RAS group, with completely unknown and unexplored functions in ruminal physiology.

In brief, the colonization of ruminal microbiota during preweaning could further influence the subsequent ruminal microbiota of adult ruminants (Ben Salem et al., 2005; Kim et al., 2016). Specifically, the effect of differentially supplementing carbohydrates in the early life of ruminants has been demonstrated (Khan et al., 2016; Meale et al., 2016). Therefore, starter feeding yak calves in early life was beneficial to the digestion and absorption function of yaks both during preweaning and in the subsequent adult period, which could be helpful for the growth of yaks.

The alteration in the ruminal metabolites we discovered as a result of feeding starter was not surprising since alteration of the ruminal microbial community generally affects the types of compounds produced by the rumen microbiota. We identified 37 different metabolites using the currently available metabolite databases.

Ruminal metabolites in pathway analysis expressed the difference in yak calves, and based on the *t*-test and FC analysis, nine pathways were revealed: chlorocyclohexane and chlorobenzene degradation, benzoate degradation, pentose and glucuronate interconversions, aminobenzoate degradation, ubiquinone, and other terpenoid-quinone biosynthesis, ABC transporters, tyrosine metabolism, pyrimidine metabolism, and microbial metabolism in diverse environment pathways. All relevant pathways were significantly upregulated in the starter feeding group due to increased concentrations of the associated metabolites xylitol and delta-tocopherol and decreased UMP and hydroquinone. This study has found that ruminants can meet up to 70% of their energy requirements and 50–70% of their protein requirements through rumen microbial metabolic activities (Wu et al., 2021). Xylitol, as an intermediate of sugar metabolism, is mainly involved in carbohydrate metabolism. In the case of the decreased insulin level in the body, xylitol is transported across the membrane to participate in the generation of liver glycogen and provide energy for the body cells (Di Rienzi and Britton Robert, 2020; Meyer-Gerspach et al., 2021). In the metabolic process of the body, xylitol is utilized by microorganisms and converted into xylulose, which enters the pentose phosphate pathway and generates short-chain fatty acids (propionate) to ensure the smooth metabolic pathway of pentose and glucuronic acid conversion (Uebanso et al., 2017). In this study, xylitol, a differential metabolite of yak calves, was significantly upregulated after supplying with starter feeding, indicating that starter feeding is helpful with propionate metabolism and thus improves the growth and development of yak calves. Hydroquinone was significantly downregulated, resulting in significant degradation of cyclochloroethane and chlorobenzene as well as benzoic acid degradation pathway, indicating that the metabolic pathway of xenobiotic degradation was significantly changed after starter feeding supplementation. UMP is a short uridine acid, consisting of uracil base, phosphate group, and ribose, it can promote the biosynthesis of glucuronic acid, uridine acid, and its derivatives play a key role in host immune response and metabolic regulation (Zhang et al., 2014). In feed production, the use of the internucleotide combination, including UMP, can promote the growth and development of animals and improve the immune function of the body (Grimble and Westwood, 2001). In this study, the metabolites of yak calves were significantly changed after the early intake of concentrate, which also caused changes in related metabolic pathways, further suggesting that early supplementation of concentrate can improve the growth performance of calves by changing rumen metabolites.

The relative abundance of *Bacteroidota* significantly decreased with the increase of DMI and showed a positive correlation with *Proteobacteria*. *Bacteroidota*, as fiber-degrading bacteria (Zapata and Quagliarello, 2015), were significantly higher in the rumen of yak calves fed only on alfalfa hay, while the nutritional monoculture and poor palatability of herbage resulted in a decrease in DMI of yak calves. *Proteobacteria* can use polysaccharides as a nutrient source for ruminants (Zimmermann, 1990), ruminal papilla length was significantly positively correlated with *Proteobacteria*, which may be due to the increased *Proteobacteria* abundance after starter feeding supplementation, which improves nutrient utilization efficiency of yak calves, promotes ruminal papilla development of yak calves, and thus improves production performance. Studies have shown that *Ruminococcus*, as a producer of carbohydrate-active enzymes (Rosewarne Carly et al., 2012), can decompose dietary fiber and improve the decomposition of hemicellulose and dietary fiber (Wang L. et al., 2019). The final body weight, DMI, and concentrations of NH_3_-N, MCP, and valerate in the rumen were proportional to the relative abundance of *Ruminococcus*, suggesting that starter feeding may improve the relative abundance of *Ruminococcus* and lead to the improvement of body growth performance.

Ruminal microbial interaction can decompose carbohydrate carbohydrates such as starch and fructose in the diet that cannot be directly absorbed by the digestive tract into monosaccharides or disaccharides. After microbial fermentation, glycogen is resynthesized and then transported to the intestine for decomposition and utilization in the small intestine, which is one of the important pathways for body function (Zhao et al., 2018). In ruminants, there are a large number of ciliates and other fiber-degrading bacteria in the rumen, and a large amount of cellulose in herbages is decomposed into short-chain fatty acids under the action of ruminal microbial fermentation, providing carbon skeleton for the synthesis of microbial proteins in the rumen and participating in the body circulation pathway (Gill and King, 2002; Zhang et al., 2017). Ruminal microbial community response changes with the change of dietary nutrient composition, which affects the types of small molecule metabolites in the digestive tract, and even threatens the immunity and health of the body (Yang and Duan, 2018). Therefore, exploring the relationship between ruminal microbiota and metabolomics is helpful to reasonably improve the diet structure and early prevention of diseases. In the correlation analysis, the metabolite UMP was positively correlated with microbial *Bacteroidota* in the rumen, and negatively correlated with *Firmicutes*. In the 16s rRNA sequencing, the relative abundance of *Bacteroidota* in yak calves fed with starter feeding significantly decreased, and the difference metabolite UMP significantly decreased, and the trend was consistent. In the functional analysis of differential metabolites, UMP was involved in the metabolism of pyrimidine in the nucleotide metabolic pathway. At the genus level, delta-tocopherol was significantly positively correlated with *Ruminococcus* and *Sharpea*, while the *Ruminococcus* was significantly increased after the yak calves were fed with starter feeding, and the metabolite delta-tocopherol was also significantly upregulated, which promoted the metabolism of coenzyme factors and vitamins. Therefore, UMP and delta-tocopherol can be used as potential markers for exploring the dominant ruminal microbiota. Studies have shown that the correlation between ruminal microbiota and metabolomics plays an important role in the treatment of ruminant ruminal acidosis and other diseases, and there is a direct or indirect correlation between microbiota candidate metabolomics (Mao et al., 2016; Xue et al., 2020). Wang changed the diet structure of dairy calves and found that the changes in the ruminal environment were caused by microbial community structure and metabolites (Wang B. et al., 2019). Pickard used high-throughput sequencing to detect the activity of intestinal microbiota and suggested that when metabolomics was associated with the abundance of microbiota, in-depth exploration of the correlation mechanism between the two had important advantages for monitoring the physiological state of the body and preventing diseases (Pickard Joseph and Chervonsky Alexander, 2015). How ruminal microbiota and metabolomics affect and associate with each other remains to be further explored.

The untargeted metabolomics approach depends on comparing peak intensity to evaluate differences in the relative abundance of metabolites with the disadvantage of a lack of accuracy and precision (Veenstra Timothy, 2012). Furthermore, identifying metabolites accurately is a tough challenge due to the complexity and chemical diversity of the metabolome (Aretz and Meierhofer, 2016). Another limitation is the small number of yak calves in similar conditions. Even with these limitations, our study enhances the understanding of the effects of starter feeding and confirms the usefulness of 16S rRNA sequencing and untargeted metabolomics analyses in ruminant nutrition studies, especially yak calves.

## Conclusion

In summary, a supplement to the starter promoted organ development and increased the abundance of some amylolytic bacteria. Ruminal microbiota have positive associations with metabolites involved in carbohydrate metabolism, nucleotide metabolism, xenobiotic biodegradation, and metabolism, demonstrating that the starter feeding can improve the nutritional status of the yak calves.

## Data Availability Statement

The original contributions presented in the study are publicly available. These data can be found here: https://dataview.ncbi.nlm.nih.gov/object/PRJNA808822, BioProject: PRJNA808822.

## Ethics Statement

The animal study was reviewed and approved by the Animal Ethics Committee of College of Agriculture and Animal Husbandry in Qinghai University. Written informed consent was obtained from the owners for the participation of their animals in this study.

## Author Contributions

YW, SL, and ZC conceived and designed the experiments. YW, ZC, SL, HX, QY, and DY mainly performed the experiments. YW and ZC analyzed the data. ZC and SL contributed the reagents, materials, and analysis tools, had primary responsibility for final content. YW and ZC wrote the manuscript. All authors read and approved the final manuscript.

## Conflict of Interest

The authors declare that the research was conducted in the absence of any commercial or financial relationships that could be construed as a potential conflict of interest.

## Publisher’s Note

All claims expressed in this article are solely those of the authors and do not necessarily represent those of their affiliated organizations, or those of the publisher, the editors and the reviewers. Any product that may be evaluated in this article, or claim that may be made by its manufacturer, is not guaranteed or endorsed by the publisher.
